# Association between Physical Activity and the Risk of Mortality and Hospitalization in Older Korean Adults with Heart Failure

**DOI:** 10.31083/j.rcm2305153

**Published:** 2022-04-26

**Authors:** Ga-In Yu, Pil-Sung Yang, Moon-Hyun Kim, Moo-Nyun Jin, Eunsun Jang, Hee Tae Yu, Tae-Hoon Kim, Hui-Nam Pak, Moon-Hyoung Lee, Boyoung Joung

**Affiliations:** ^1^Division of Cardiology, Department of Internal Medicine, Yonsei University College of Medicine, 03722 Seoul, Republic of Korea; ^2^Division of Cardiology, CHA Bundang Medical Center, CHA University, 13497 Seongnam, Republic of Korea; ^3^Division of Cardiology, Department of Internal Medicine, Sangye Paik Hospital, Inje University College of Medicine, 01757 Seoul, Republic of Korea

**Keywords:** exercise, heart failure, physical activity

## Abstract

**Background:**

Regular exercise improves the functional ability and quality of life of patients with heart failure (HF). However, studies on the results of intensity of exercise in the older population are scarce, especially in the Asian population.

**Method and Results:**

A total of 8982 older people (age ≥65 years) with HF were selected from the Korean National Health Insurance Service-Senior database (2005–2012). Participants were stratified according to the levels of physical activity per week as follows: (1) inactive group; (2) insufficiently active group: 1–499 metabolic equivalent task minutes (MET-min)/week; (3) active group: 500–999 MET-min/week; and (4) highly active group: ≥1000 MET-min/week. During a median follow-up period of 3.2 years, the incidence and risk of mortality were reduced in the insufficiently active (6.7 vs. 4.2 per 100 person-years, adjusted hazard ratio [HR], 0.82; 95% confidence interval [CI], 0.71–0.94; *p *< 0.001), active (3.8 per 100 person-years; HR, 0.81; 95% CI, 0.70–0.95; *p* = 0.010), and highly active (2.4 per 100 person-years; HR, 0.52; 95% CI, 0.41–0.67; *p *< 0.001) groups compared to inactive patients.

**Conclusions:**

In older Asians with HF, increased physical activity reduced the risk of all-cause mortality. The mortality-reducing benefit started at a lower physical activity compared to the World Health Organization guideline (500–999 MET-min/week), and the risk decreased with more physical activity.

## 1. Introduction

Heart failure (HF) has become an important public medical concern due to an increase in aging population. HF causes socioeconomic burdens on individuals and society [1]. It also has high morbidity and mortality rates, with approximately 30–40% of patients dying within 1 year after being diagnosed with HF [2]. The prevalence of HF in Asia is reported to range between 1.2% and 6.7%, depending on the study population [3, 4].

Physical activity reduces not only vascular diseases but also nonvascular morbidity as well as the mortality [5]. Recent guidelines recommend moderate-intensity physical activity (MPA); 150–300 min/week and vigorous-intensity physical activity (VPA); 75–150 min/week, which translates to 500–999 metabolic equivalent task min/week (MET-min/week) in population 65 years or older [6, 7]. Completing >150 min/week of MPA exercise (i.e., brisk walking or other moderate-intensity aerobic activities) reduces the risk of morbidity, mortality, disability, and frailty by more than 30% compared to inactivity [8, 9].

The effectiveness of exercise in HF patients is based on studies that actively implemented exercise-training programs within a safe range of exercise intensity in stable patients undergoing adequate medical treatment [10]. Meta-analyses showed that exercise can reduce the mortality risk in patients with HF, and these results were maintained even with short-term exercise (under 12 months) [11, 12, 13].

However, studies on the Asian population are still lacking and in particularly, although the prevalence of heart failure is high, there are not many results according to exercise intensity in the elderly population who has difficulty in high-intensity exercise. The aim of this study was to determine the effect of intensity of physical activity on the mortality risk in older individuals (age ≥65 years) with HF in the Asian population. We also evaluated other clinical outcomes, including all-cause hospitalizations, HF-related hospitalizations, cardiovascular disease (CVD), and stroke.

## 2. Methods

### 2.1 Study Population

Data were collected from the National Health Insurance Service of Korea (NHIS)-Senior database, which included data of 312,736 populations collected via a 10% simple random sampling method from a total of 5,500,000 populations 65 years or older in the National Health Information Database [14, 15]. The NHIS-Senior database included socioeconomic and insurance status, medical history, and check-up test results.

**Fig. 1.**
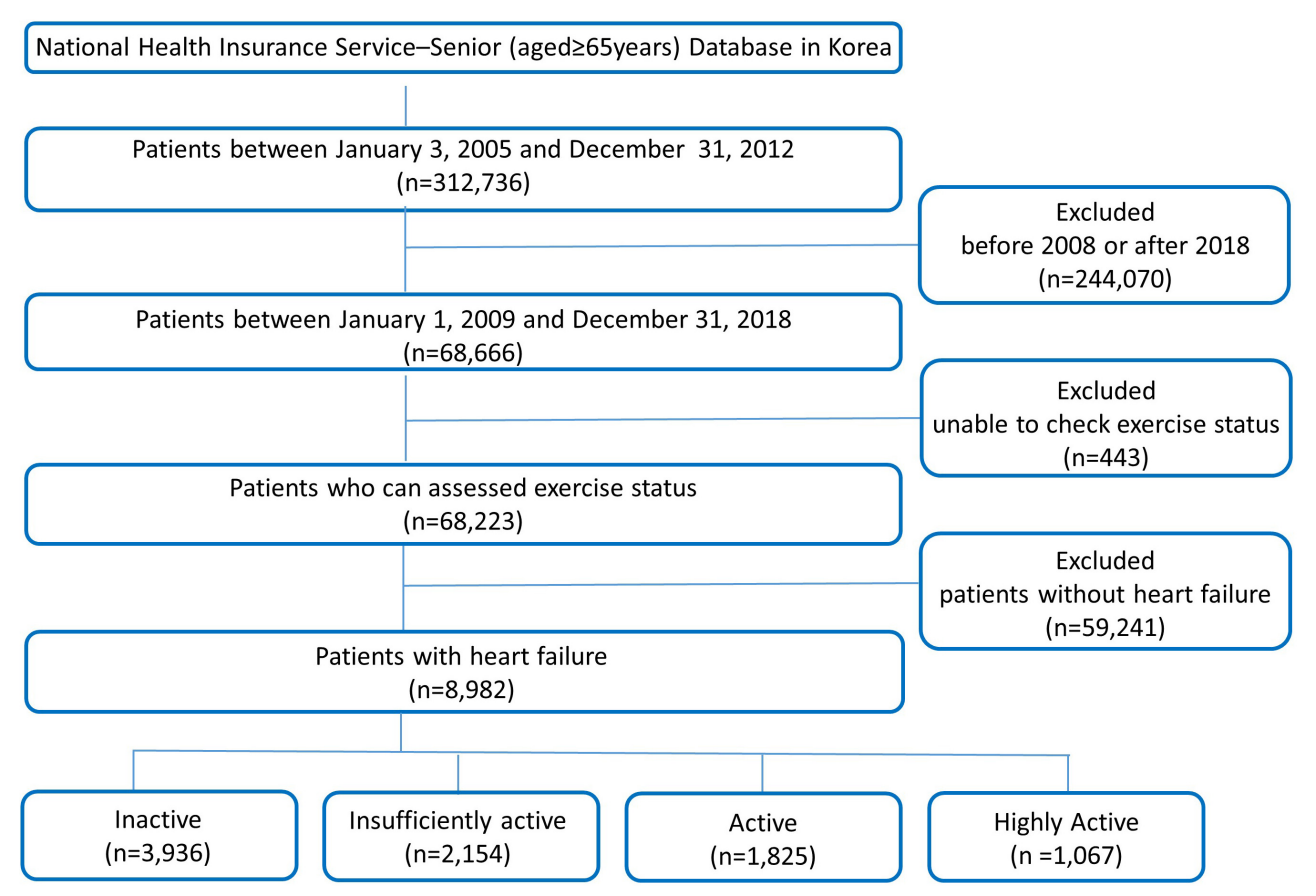
**Flow diagram of the study population**. A total of 8982 heart failure patients aged 65 years or older in Korea were classified according to exercise intensity.

This study included the data of 8982 older adults (age ≥65 years) with a history of HF from the Korean NHIS-Senior database, for whom health check-up data were available between 2009 and 2013 and who were followed up until December 2015 (Fig. 1). Criteria of HF in this study was based on ICD-10 codes (I11.0, I50, and I97.1) for one inpatient or two outpatient visit in the database (**Supplementary Table 1**).

### 2.2 Assessment of Physical Activity Level

The level of physical activity during waking time was evaluated through a self-report questionnaire using the 7-day recall method [16, 17, 18]. The questionnaire asked about frequency (days/week) of the following three intensity levels: (i) low-intensity physical activity (LPA) for at least 30 min, (ii) MPA for at least 30 min, and (iii) VPA for at least 20 min. LPA include slow speed of walking, MPA include like normal speed of walking and cycling and VPA include high speed of running and cycling.

Using the Ainsworth *et al*. [19] compendium, 3.3 METs for LPA, 4.0 METs for MPA, and 8.0 METs for VPA were assigned. And the total physical activity-related energy expenditure (MET-min/week) was the sum of duration frequency and intensity of exercise [19]. The participants were stratified according to their total weekly levels of physical activity as follows: (1) highly active group: physical activity exceeding the guideline target range (≥1000 MET-min/week); (2) active group: physical activity within the guideline target range (500–999 MET-min/week); (3) insufficiently active group: physical activity less than the guideline target range (1–499 MET-min/week); and (4) inactive group: no physical activity other than minimal movement in their spare time, considering the guidelines and previous studies [6, 7, 20].

### 2.3 Baseline Comorbidities

Baseline comorbidities were assessed with the ICD-10 codes and drug prescription data (**Supplementary Table 1**). To ensure diagnostic accuracy, as in previous studies, if the diagnosis was registered at the time of discharge or the same diagnosis was registered twice or more in an outpatient clinic, the case was considered a corresponding comorbidity [15, 21, 22]. We performed analysis of drug history for heart failure. Among ACE (angiotensin-converting enzyme) inhibitor or ARB (angiotensin II receptor blocker), BB (beta blocker), loop diuretics and mineralocorticoid receptor antagonist, HF medication group I was assumed if no medications were taken or only ACE inhibitors or ARBs were taken. HF medication group II was assumed if BB or loop diuretics were taken but not mineralocorticoid receptor antagonist, and HF medication group III for patients taking mineralocorticoid receptor antagonist [23].

### 2.4 Outcomes

The primary outcome was all-cause mortality. Information on death (cause and time) was checked through a unique identification number in the demographics of the National Statistical Office [15, 22]. Since the NHIS is a national institution for all Koreans, this approach was used to ensure event-related information. And the mortality was analyzed by dividing them into CVD-related death and non-CVD-related death.

The secondary outcome was all-cause hospitalization. The hospitalization cause was based on the diagnosis at the time of discharge. Eligible causes of hospitalization such as exacerbation of HF, CVD, chronic ischemic heart disease, coronary vascular disease, and ischemic stroke were included. The accuracy of diagnosis based on NHIS claims data has already been verified [22]. The details of the clinical outcomes are described in **Supplementary Table 1**. Follow-ups of mortality and hospitalization were conducted until December 2015.

### 2.5 Statistical Analysis

Descriptive statistics were used to analyze the baseline characteristics of participants. Categorical variables are expressed as ratios (percentages), and continuous variables are expressed as median and interquartile range. Fisher’s test or chi-square test was used for comparison between categorical variables, and Student’s *t*-test was used for comparison between continuous variables. The incidence of each clinical outcome was expressed as number of events/100 person-years.

Competing risk regression for all-cause death events was carried out with the Fine-Gray method. Multivariable regressions were carried out with adjustment for age, gender, hypertension, diabetes, dyslipidemia, vascular disease, stroke, renal disease, lung disease, cancer, body mass index, smoking and drinking. Additionally, investigate the effects of physical activity as continuous value (0 MET-min/week as reference) on mortality via cubic spline curve.

All test with values of *p *< 0.05 was considered statistically significant. Statistical analyses were performed using R programming version 4.0.3 (The R Foundation for Statistical Computing, Vienna, Austria).

## 3. Results

### 3.1 Baseline Characteristics

A total of 8982 individuals 65 years or older (mean age, 75.5 ± 6.3 years) with HF were included in the analysis. The comparison of baseline characteristics among groups classified by activity level are summarized in Table 1. The participants were divided into inactive, insufficiently active, active, and highly active groups, accounting for 15.6%, 13.0%, 11.4%, and 10.2% of the study population, respectively.

**Table 1.** **Patients’ baseline characteristics by physical activity level**.

		Inactive	Insufficiency active	Active	Highly active	*p* value
		(N = 3936)	(N = 2154)	(N = 1825)	(N = 1067)	
Demographic						
	Age, years	76.9 ± 6.7	75.2 ± 6.0	74.1 ± 5.5	73.2 ± 5.0	<0.001
	Male	927 (23.6%)	577 (26.8%)	633 (34.7%)	508 (47.6%)	<0.001
	Body mass index, kg/$m^{2}$	24.0 ± 3.9	24.6 ± 3.7	24.5 ± 3.4	24.8 ± 3.2	<0.001
	Waist, cm	83.7 ± 10.0	85.0 ± 9.3	84.7 ± 8.6	85.8 ± 8.5	<0.001
	Systolic blood pressure, mmHg	130.7 ± 18.2	130.5 ± 16.9	131.8 ± 17.0	132.4 ± 17.5	0.005
	Diastolic blood pressure, mmHg	77.8 ± 10.8	77.7 ± 10.7	78.0 ± 10.5	78.5 ± 10.8	0.243
	Smoking	549 (13.9%)	378 (17.5%)	403 (22.1%)	329 (30.8%)	<0.001
	Alcohol	305 (7.7%)	274 (12.7%)	293 (16.1%)	254 (23.8%)	<0.001
Risk scores						
	Hospitality frailty risk score	5.3 ± 7.5	3.3 ± 5.1	2.7 ± 4.3	2.4 ± 4.4	<0.001
	Charlson comorbidity index	5.7 ± 2.9	5.6 ± 3.0	5.4 ± 2.8	5.3 ± 2.9	<0.001
Comorbidities						
	Hypertension	3633 (92.3%)	2000 (92.9%)	1696 (92.9%)	991 (92.9%)	0.774
	Diabetes mellitus	1268 (32.2%)	710 (33.0%)	613 (33.6%)	362 (33.9%)	0.628
	Dyslipidemia	2783 (70.7%)	1579 (73.3%)	1393 (76.3%)	813 (76.2%)	<0.001
	Chronic kidney disease	298 (7.6%)	146 (6.8%)	122 (6.7%)	65 (6.1%)	0.290
	Vascular disease	1114 (28.3%)	577 (26.8%)	503 (27.6%)	302 (28.3%)	0.619
	Ischemic stroke or TIA	1461 (37.1%)	716 (33.2%)	600 (32.9%)	317 (29.7%)	<0.001
	COPD	1062 (27.0%)	580 (26.9%)	393 (21.5%)	229 (21.5%)	<0.001
	Malignancy	793 (20.1%)	489 (22.7%)	376 (20.6%)	236 (22.1%)	0.094
	Osteoporosis	2362 (60.0%)	1203 (55.8%)	899 (49.3%)	477 (44.7%)	<0.001
	Previous MI	577 (14.7%)	269 (12.5%)	221 (12.1%)	130 (12.2%)	0.012
	Peripheral artery disease	697 (17.7%)	378 (17.5%)	334 (18.3%)	205 (19.2%)	0.638
Laboratory findings						
	Fasting blood glucose, mmol/L	109.0 ± 38.8	109.0 ± 35.5	110.1 ± 36.7	111.9 ± 39.5	0.135
	Total cholesterol, mg/dL	189.7 ± 42.7	190.8 ± 40.9	189.4 ± 41.7	187.5 ± 40.3	0.220
	Triglyceride, mg/dL	143.8 ± 80.3	146.4 ± 84.4	143.3 ± 83.8	143.8 ± 89.4	0.624
	LDL-cholesterol, mg/dL	111.2 ± 38.9	110.3 ± 36.6	109.3 ± 36.7	107.4 ± 36.5	0.025
	HDL-cholesterol, mg/dL	51.1 ± 28.9	51.6 ± 18.9	52.0 ± 24.5	51.3 ± 13.5	0.657
	AST, U/L	25.5 ± 14.6	26.7 ± 23.1	26.9 ± 19.1	26.5 ± 11.9	0.017
	ALT, U/L	20.2 ± 15.4	21.8 ± 13.6	22.8 ± 26.1	22.7 ± 14.6	<0.001
	Gamma GT, U/L	31.8 ± 45.1	34.6 ± 49.8	35.4 ± 53.1	37.8 ± 45.9	0.001
	Serum creatinine, mg/dL	1.1 ± 1.1	1.1 ± 1.1	1.1 ± 0.9	1.1 ± 3.1	0.798
	eGFR, mL/min/1.73 $m^{2}$	63.8 ± 20.5	65.1 ± 19.8	66.4 ± 19.3	68.5 ± 18.5	<0.001
Medication history						
	Aspirin	3093 (78.6%)	1682 (78.1%)	1426 (78.1%)	831 (77.9%)	0.944
	P2Y12 inhibitor	1214 (30.8%)	619 (28.7%)	547 (30.0%)	321 (30.1%)	0.400
	Vitamin K antagonist	318 (8.1%)	181 (8.4%)	147 (8.1%)	87 (8.2%)	0.972
	NOAC	15 (0.4%)	9 (0.4%)	12 (0.7%)	6 (0.6%)	0.460
	ACE inhibitors or ARB	3111 (79.6%)	1714 (79.6%)	1446 (79.2%)	833 (78.1%)	0.800
	Beta-blockers	2680 (68.1%)	1466 (68.1%)	1228 (67.3%)	737 (69.1%)	0.801
	Diuretics	3566 (90.6%)	1910 (88.7%)	1599 (87.6%)	922 (86.4%)	<0.001
	Mineralocorticoid receptor antagonist	1643 (41.7%)	812 (37.7%)	642 (35.2%)	346 (32.4%)	<0.001
	Statin	1897 (48.2%)	1074 (49.9%)	941 (51.6%)	571 (53.5%)	0.007

Values are presented as mean ± standard deviation or n (%). ALT, alkaline phosphatase; AST, aspartate aminotransferase; COPD, chronic obstructive pulmonary disease; eGFR, estimated glomerular filtration rate; HDL, high-density lipoprotein; LDL, low-density lipoprotein; MI, myocardial infarction; TIA, transient ischemic attack; NOAC, Non vitamin K antagonist oral anticoagulant; ACE, angiotensin-converting enzyme; ARB, angiotensin II receptor blocker; HF, heart failure.

### 3.2 Level of Physical Activity and Mortality

**Fig. 2.**
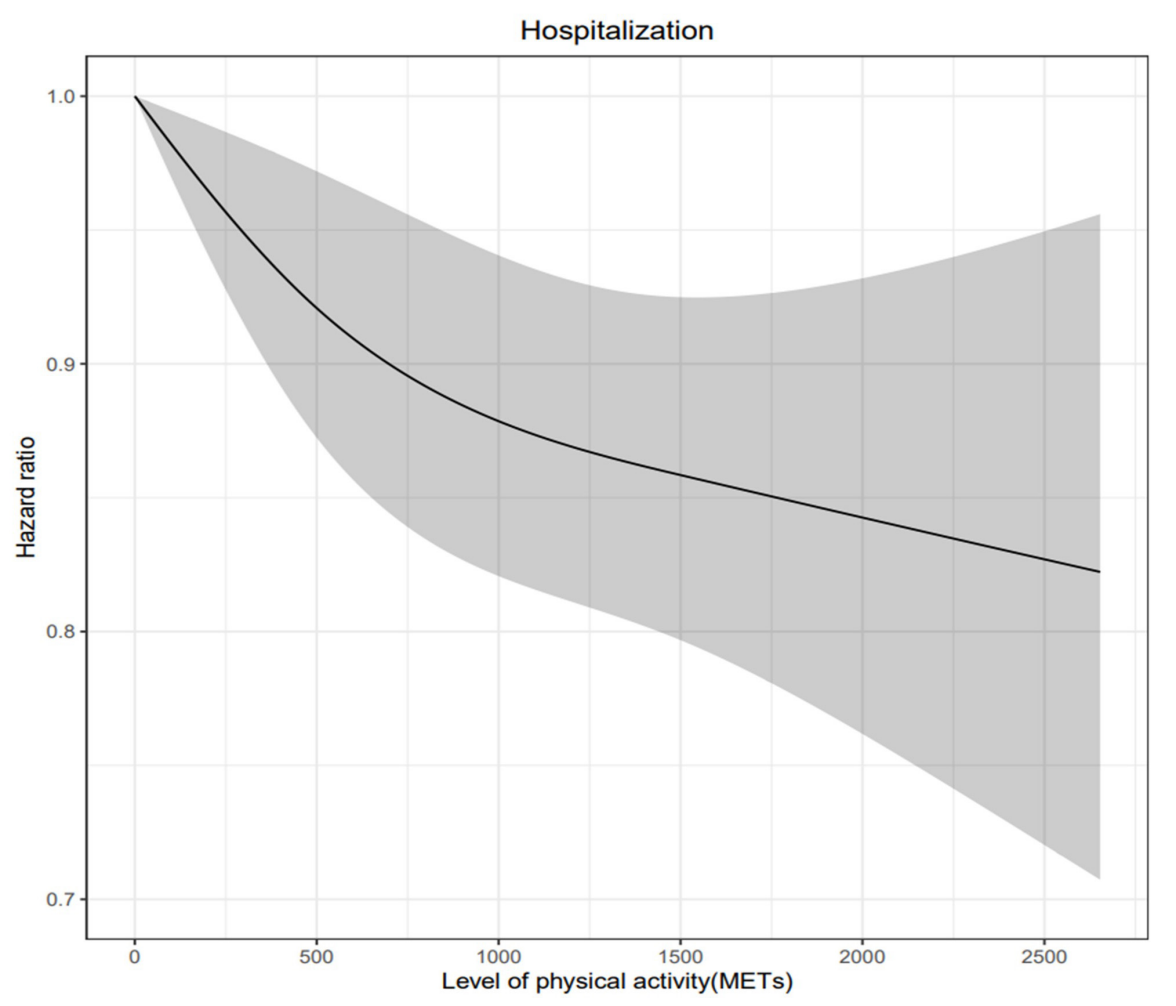
**Weekly level of physical activity and adjusted hazard ratio for all-cause hospitalization in older adults with heart failure**. MET, metabolic equivalent task. Non-linear cubic spline curve of weekly physical activity level against hospitalization. Black line represents the fitted line of the association between weekly physical activity level and adjusted hazard ratio of hospitalization, whereas the shaded region represents the 95% confidence interval.

During the mean follow-up period of 92.8 ± 36.9 months, 1359 (15.1%) participants had all-cause mortality with an overall incidence of 5.0/100 person-years. The all-cause mortality rates were 6.7, 4.2, 3.8, and 2.4/100 person-years in the inactive, insufficiently active, active, and highly active groups, respectively (Table 2). In the Fine-Gray competing risk multivariable regression analysis, the insufficiently active (HR, 0.82; 95% CI, 0.71–0.94), active (HR, 0.81; 95% CI, 0.70–0.95), and highly active (HR, 0.52; 95% CI, 0.41–0.67) groups were showed lower risks of mortality compared to the inactive group. Fig. 2 presents the risk of hospitalization associated with continuous values of level of physical activity with a cubic spline curve. The risk of mortality associated with continuous values of level of physical activity with a cubic spline curve. There was a linear relationship between physical activity and risk of mortality. As the level of physical activity increased, the mortality risk was further reduced.

**Table 2.** **Risk of all-cause mortality, cardiovascular mortality, and non-cardiovascular disease related mortality according to physical activity among older adults with heart failure**.

		Patients (n)	Events (n)	Events/100 PYR	Unadjusted HR (95% CI)	*p* value	Adjusted HR (95% CI)	*p* value
All-cause mortality								
	Inactive	3936	776	6.7	Reference		Reference	
	Insufficiently active	2154	283	4.2	0.63 (0.55–0.72)	<0.001	0.82 (0.71–0.94)	0.010
	Active	1825	218	3.8	0.56 (0.48–0.65)	<0.001	0.81 (0.70–0.95)	0.010
	Highly active	1067	82	2.4	0.35 (0.28–0.44)	<0.001	0.52 (0.41–0.67)	<0.001
CVD mortality								
	Inactive	3936	261	2.2	Reference		Reference	
	Insufficiently active	2154	95	1.4	0.63 (0.50–0.79)	<0.001	0.90 (0.70–1.14)	0.370
	Active	1825	77	1.3	0.59 (0.46–0.76)	<0.001	0.94 (0.72–1.24)	0.670
	Highly active	1067	30	0.9	0.38 (0.26–0.56)	<0.001	0.68 (0.45–1.01)	0.060
Non-CVD-related mortality								
	Inactive	3936	515	4.5	Reference		Reference	
	Insufficiently active	2154	188	2.8	0.63 (0.53–0.74)	<0.001	0.78 (0.65–0.93)	0.010
	Active	1825	141	2.5	0.55 (0.45–0.66)	<0.001	0.75 (0.62–0.91)	<0.001
	Highly active	1067	52	1.5	0.33 (0.25–0.44)	<0.001	0.46 (0.34–0.62)	<0.001

HR, hazard ratio; CI, confidence interval; CVD, cardiovascular disease; PYR, person-years at risk. The model was adjusted for age, sex, body mass index, hypertension, diabetes, dyslipidemia, chronic kidney disease, chronic obstructive pulmonary disease, malignancy, previous myocardial infarction, peripheral artery disease, vascular disease, prior stroke or transient ischemic attack, osteoporosis, Hospital Frailty Risk Score, Hospital Frailty Risk Score category, Charlson Comorbidity Index, smoking, and alcohol drinking.

The incidence of non-CVD-related mortality was 4.5, 2.8, 2.5, and 1.5/100 person-years in the inactive, insufficiently active, active, and highly active groups, respectively. Compared to the inactive group, the adjusted risk of non-CVD-related mortality was reduced in the insufficiently active (HR, 0.78; 95% CI, 0.65–0.93), active (HR, 0.75; 95% CI, 0.62–0.91), and highly active (HR, 0.46; 95% CI, 0.34–0.62) groups. However, the adjusted risk of cardiovascular-related mortality was not reduced by physical activity (Table 2).

### 3.3 Level of Physical Activity and Hospitalization

During the follow-up period, 5788 patients (64.4%) were hospitalized. The overall incidence of all-cause hospitalizations during follow-up was 35.8/100 person-years. When divide the patients according to their level of intensity of physical activity, the incidence rates were 39.8, 35.9, 33.1, and 27.9 cases/100 person-years in the inactive, insufficiently active, active, and highly active groups, respectively (Table 3). In the Fine-Gray competing risk multivariable regression models, compared with the inactive group, the insufficiently active (HR, 0.98; 95% CI, 0.91–1.04), active (HR, 0.94; 95% CI, 0.88–1.01), and highly active (HR, 0.81; 95% CI, 0.74–0.89) groups were showed a lower risk of hospitalization. Fig. 2 presents the risk of hospitalization associated with continuous values of level of physical activity with a cubic spline curve. A non-linear relationship between physical activity and the risk of hospitalization was evident. Although the risk of hospitalization was further reduced with increased level of physical activity, the benefit of increased physical activity did not increase beyond 1500 MET-min/week.

**Table 3.** **Risk of hospitalization due to all-cause and heart failure, cardiovascular disease and stroke according to physical activity among older adults with heart failure**.

		Patients (n)	Events (n)	Events/100 PYR	Unadjusted HR (95% CI)	*p* value	Adjusted HR (95% CI)	*p* value
Hospitalization due to all-cause								
	Inactive	3936	2613	39.8	Reference		Reference	
	Insufficiently active	2154	1400	35.9	0.91 (0.85–0.97)	0.004	0.98(0.91–1.04)	0.490
	Active	1825	1156	33.1	0.84 (0.79–0.90)	<0.001	0.94(0.88–1.01)	0.110
	Highly active	1067	619	27.9	0.72 (0.66–0.79)	<0.001	0.81(0.74–0.89)	<0.001
Hospitalization due to heart failure								
	Inactive	3936	287	2.6	Reference		Reference	
	Insufficiently active	2154	146	2.3	0.88 (0.72–1.08)	0.216	1.04 (0.85–1.27)	0.740
	Active	1825	107	1.9	0.75 (0.60–0.93)	0.010	0.96 (0.77–1.21)	0.750
	Highly active	1067	49	1.4	0.56 (0.42–0.76)	<0.001	0.75 (0.54–1.02)	0.070
Cardiovascular disease								
	Inactive	3936	159	1.4	Reference		Reference	
	Insufficiently active	2154	79	1.2	0.86 (0.66–1.13)	0.273	0.92 (0.70–1.21)	0.550
	Active	1825	78	1.4	0.99 (0.76–1.30)	0.939	1.04 (0.78–1.38)	0.790
	Highly active	1067	51	1.5	1.08 (0.79–1.48)	0.633	1.11 (0.80–1.54)	0.520
Stroke								
	Inactive	3936	246	2.2	Reference		Reference	
	Insufficiently active	2154	135	2.1	0.95 (0.77–1.17)	0.643	1.02 (0.82–1.26)	0.870
	Active	1825	108	1.9	0.87 (0.70–1.10)	0.242	0.93 (0.73–1.17)	0.520
	Highly active	1067	50	1.5	0.67 (0.49–0.91)	0.009	0.70 (0.51–0.96)	0.030

HR, hazard ratio; CI, confidence interval; PYR, person-years at risk. The model was adjusted for age, sex, body mass index, hypertension, diabetes, dyslipidemia, chronic kidney disease, chronic obstructive pulmonary disease, malignancy, previous myocardial infarction, peripheral artery disease, vascular disease, prior stroke or transient ischemic attack, osteoporosis, Hospital Frailty Risk Score, Hospital Frailty Risk Score category, Charlson Comorbidity Index, smoking, and alcohol drinking.

Compared to the inactive group, the incidence and risk of stroke were reduced in the highly active group (2.2 vs. 1.5/100 person-years; HR, 0.70; 95% CI, 0.51–0.96; *p* = 0.030) (Table 3). However, the risks of HF admission and CVD were not reduced by increased physical activity.

### 3.4 Subgroup Analysis

**Fig. 3.**
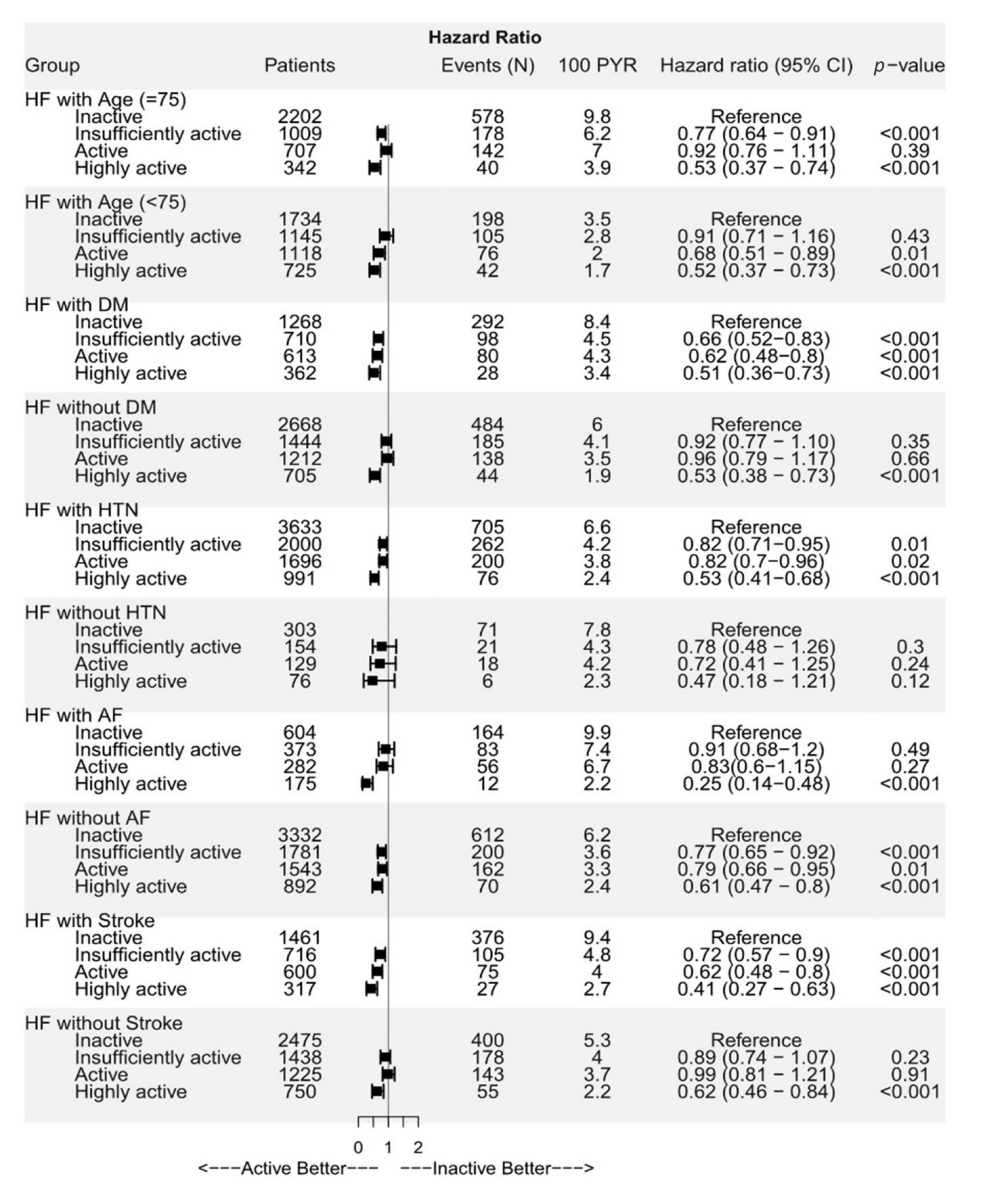
**Subgroup analyses of all-cause mortality in older adults with heart failure**. AF, atrial fibrillation; CI, confidence interval; DM, diabetes mellitus; HF, heart failure; HTN, hypertension; PYR, person-years at risk. Forest plot showing the adjusted hazard ratios of all-cause death in older (age ≥65 years) patients with heart failure. In all cases of HF, regardless of the presence or absence of DM, HTN, AF, and stroke, exercise reduced all-cause mortality. Even minimal exercise reduced all-cause death compared to no exercise, and the degree of mortality reduction increased as the level of exercise increased.

The risk of mortality was assessed in different patient subgroups. A risk reduction of mortality was observed regardless of the patient’s age, sex, hypertension, diabetes, chronic kidney disease, and previous history of stroke (Fig. 3). In subgroup analysis according to gender, the adjusted risk of all-cause mortality was reduced in the insufficiently active (in female: HR, 0.81; 95% CI, 0.67–0.97 and in male: HR, 0.81; 95% CI, 0.64–1.03), active (in female: HR, 0.79; 95% CI, 0.64–0.99 and in male: HR, 0.85; 95% CI, 0.68–1.07), and highly active (in female: HR, 0.58; 95% CI, 0.40–0.84 and in male: HR, 0.50; 95% CI, 0.36–0.68) groups (**Supplementary Table 2**). The adjusted risk of all-cause hospitalization was presented at **Supplementary Table 3**.

In a subgroup analysis of the HF medication group, for all-cause mortality and non-CVD-related mortality, there was no difference in outcome with increasing exercise in HF medication group I—none or ACE inhibitor/ARB, but all-cause mortality and non-CVD-related mortality were decrease with increasing exercise in HF medication group II—beta blocker or diuretics, III—spironolactone (**Supplementary Table 4**). On the other hand, in the case of hospitalization due to heart failure and cardiovascular disease, exercise had a positive effect on outcome in HF medication group I—none or ACE inhibitor/ARB, but in HF medication group II—beta blocker or diuretics, III—spironolactone, there was no significant correlation between exercise change and outcome (**Supplementary Table 5**).

## 4. Discussion

This large scale, real-world nationwide cohort study of older Asians with HF demonstrated the following results. First, known effects of exercise on HF were reconfirmed in an older Asian population. Increased level of physical activity decreased the risk of all-cause mortality. In particular, the risk of non-CVD mortality (vs. CVD-related mortality) was significantly reduced with increased physical activity. Moreover, increased physical activity also reduced the risk of hospitalization in older patients with HF. Second, and more importantly, the effect of decreasing mortality with more physical activity started at level lower of physical activity compared to the World Health Organization (WHO) guideline of 500–999 MET-min/week.

### 4.1 Physical Activity and Mortality in Older Adults with HF

The recent Cochrane review of exercise training [12], including 33 trials in 4740 patients with HF (primarily HF with a reduced ejection fraction), showed that exercise tends to reduce mortality in trials using 1 year of follow-up. Compared to the control group, the exercise-trained group had lower hospitalization rates (both all-cause and HF-related hospitalizations) and improved the quality of life of patients. Practical recommendations for exercise training have been published by the Heart Failure Association [24]. Also, a single large-scale randomized controlled trial showed a modest decrease in the primary composite outcome of all-cause mortality or all-cause hospitalization [25]. However, this study did not show a significant reduction in mortality [25, 26]. Therefore, although the importance of exercise in HF has been proven, there is insufficient information on the results of exercise intensity, with limited number of related studies, especially on Asians.

### 4.2 Dose of Physical Activity and Mortality in Older Adults with HF

**Fig. 4.**
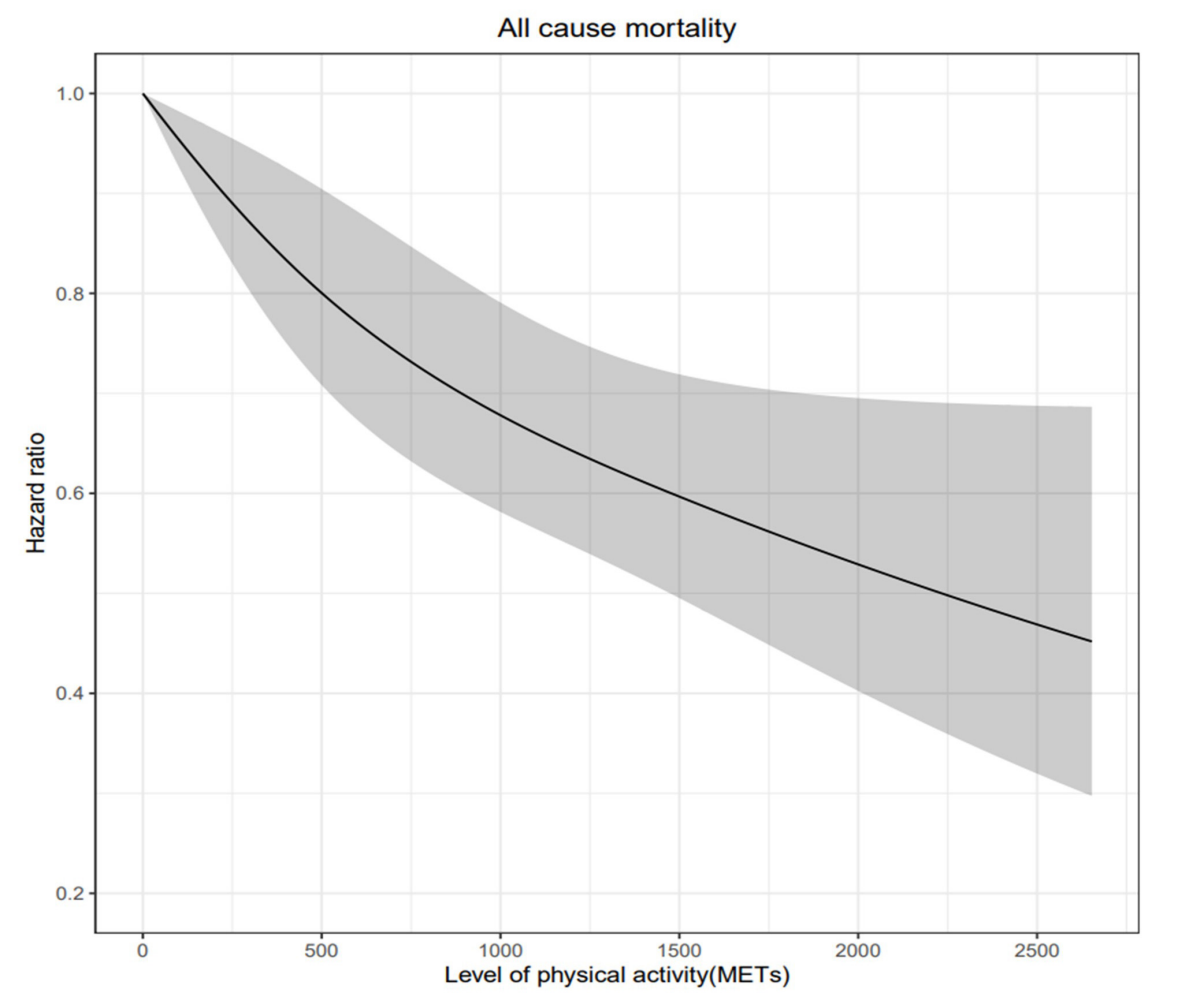
**Adjusted hazard ratio for all-cause mortality in older adults with heart failure by weekly physical activity levels**. MET, metabolic equivalent task. Non-linear cubic spline curve of physical activity level against all-cause mortality. Black line represents the fitted line of the association between weekly physical activity level and adjusted hazard ratio of mortality, whereas the shaded region represents the 95% confidence interval.

As the life expectancy of HF peoples increases, comprehensive medical support for them, including lifestyle modifications, such as exercise, has become increasingly important. In this study, the dose-response relationship between physical activity and mortality was evaluated by calculating the total duration physical activity as a continuous variable. The positive benefits of increased physical activity on mortality started to appear at lower levels of activity (Fig. 4). These results showed that little activity is better than no activity at all.

Also, the same results were confirmed in the analysis by gender difference. Although many studies demonstrated that physical activity and physical exercise may prevent negative cardiovascular adaptations associated with the postmenopausal period, which in turn could lead to the development of heart failure [27, 28], this study showed that physical activity reduced all-cause mortality and all-cause hospitalization even in elderly females with HF.

In the past, exercise was performed for the prevention of diseases including heart failure and it is already known that exercise has therapeutic implications [29, 30]. This is also specified in current treatment guidelines [7]. This study showed that the therapeutic effect of such exercise was also applied to Asian, older adults, and heart failure patients, and that even lower-intensity exercise than the guideline was meaningful.

### 4.3 Limitations

This study had limitations. First, it did not include echocardiographic data, such as left ventricular ejection fraction, or assessment of symptoms, such as the New York Heart Association Functional Classification, since the diagnostic criteria of HF were based on the ICD codes. Although we presented the outcome according to the different degree of medication for heart failure, the severity of HF could not be evaluated. This can be an important limitation of this study, as severe HF condition can reduce physical activity. Further studies based on the severity of HF are required. Second, such studies using administrative databases could be susceptible to errors arising from coding inaccuracies. To minimize this problem, we applied the definitions that were already validated in previous studies using the Korean NHIS cohort [15, 20, 21, 22, 23, 24, 25, 26, 27, 28, 29, 30, 31]. Third, CV-related information was relatively well known in NHIS data, but it was difficult to identify all other causes of death. Therefore, we only divided total deaths into two categories: CVD-related and non-CVD-related deaths. Fourth, this study relied on self-reported data at a single point in time. Therefore, questionnaire answers may not reflect the overall patient’s condition. Fifth, although physical activity plays an important role in reducing CVD-related incidents, recent data showed that activity alone is not enough to reduce the risk of CVD in older adults [32, 33]. In this study, other factors such as sedentary behavior were not considered; therefore, additional research on these factors in the elderly Asian population is required. Despite these limitations, this study was still significant in that it was a large-sized study using physical activity data in older Asian population with HF. Herein, we assessed the correlation between exercise intensity and clinical outcomes in HF patients. Overall, our analysis may serve as the first step in determining the optimal exercise intensity for older adults with HF.

## 5. Conclusions

In older population with HF, increased level of physical activity was reduced risk of all-cause mortality. The benefit of increased physical activity on mortality began at a lower physical activity level compared to the WHO guideline of 500–999 MET-min/week, and the risk reduction of all-cause death increased with increased level of physical activity.

## Supplementary Material

Supplementary material associated with this article can be found, in the online version, at https://doi.org/10.31083/j.rcm2305153.
