# Supplementary material for: Association between Physical Activity and the Risk of Mortality and Hospitalization in Older Korean Adults with Heart Failure
Source: Rev Cardiovasc Med. 2022 Apr 26;23(5):153. doi: 10.31083/j.rcm2305153 (PMC11273939; doi:10.31083/j.rcm2305153)
Supplement: Supplementary file 1 [file 2153-8174-23-5-153-s1.zip › 2153-8174-23-5-153-s1.docx]

Supplementary Table 1. Definitions and ICD-10 codes used for defining the comorbidities and clinical outcomes.

|  | Definitions | ICD-10 codes or conditions |
| --- | --- | --- |
| Comorbidities |  |  |
| Atrial fibrillation^1-3^ | Defined from diagnosis* | ICD-10: I48 |
| Heart failure^2,3^ | Defined from diagnosis* | ICD-10: I11.0, I50, I97.1 |
| Hypertension^2,3,4^ | Defined from diagnosis* | ICD-10: I10, I11, I12, I13, I15 and antihypertensive medication |
| Diabetes mellitus^2,3^ | Defined from diagnosis* plus treatment | ICD-10: E10, E11, E12, E13, E14  Treatment: all kinds of oral antidiabetics and insulin. |
| Dyslipidemia^2,3^ | Defined from diagnosis* | ICD-10: E78 |
| Ischemic stroke^2,3^ | Defined from diagnosis* | ICD-10: I63, I64 |
| Transient ischemic attack^2,3^ | Defined from diagnosis* | ICD-10: G45 |
| Hemorrhagic stroke | Defined from diagnosis* | ICD-10: I60, I61, I62 |
| Myocardial infarction^5^ | Defined from diagnosis* | ICD-10: I21, I22, I25.2 |
| Coronary heart disease | Defined from a history of acute myocardial infarction, coronary revascularization, or chronic ischemic heart disease. | Acute myocardial infarction: admission diagnosis (ICD-10: I21, I22) concurrently with coronary angiography (HA670, HA680, HA681)  Coronary revascularization: percutaneous coronary intervention (M6551, M6552, M6561, M6563, M6562, M6564, M6571, M6572), thrombolytic treatment (M6634), or coronary artery bypass graft (O1641, OA641, O1642, OA642, O1647, OA647)  Chronic ischemic heart disease: diagnosis* (ICD-10: I25.2, I25.5, I25.6, I25.8, I25.9) |
| Peripheral arterial disease^2,3^ | Defined from diagnosis* | ICD-10: I70.0, I70.1, I70.2, I70.8, I70.9 |
| Chronic kidney disease^2,3^ | Defined from eGFR or diagnosis*  (if laboratory value was not available, diagnosis code was used) | eGFR <60mL/min per 1.73 m^2^  ICD-10: N18, N19 |
| End-stage renal disease^6^ | Defined from national registry for severe illness. | Patients with end-stage renal disease undergoing chronic dialysis or received a kidney transplant. |
| Hypertrophic cardiomyopathy^7^ | Defined from at least one records of either inpatient or outpatient diagnoses | ICD-10: I42.1, I42.2 |
| Sleep apnea | Defined from diagnosis* | ICD-10: G47.3 |
| Proteinuria | Defined from laboratory data (if laboratory value was not available, diagnosis code was used) | Urine dipstick proteinuria 1+ or higher (ICD-10: N06, N391, N392, R80) |
| Osteoporosis^8^ | Defined from diagnosis* | ICD-10: M80, M81, M82 (except M82.0) |
| Hyperthyroidism | Defined from diagnosis* | ICD-10: E05 |
| Hypothyroidism | Defined from diagnosis* | ICD-10: E03 |
| Chronic Liver disease | Defined from diagnosis of chronic liver disease, cirrhosis, and hepatitis | ICD-10: B18, K70, K71, K72, K73, K74, K76.1 |
| Chronic obstructive pulmonary disease^9^ | Defined from diagnosis* plus treatment | ICD-10: J42, J43(except J43.0), J44  Treatment: SABA, SAMA, LABA, LAMA, ICS, ICS+LABA, or methylxanthine (>1 months). |
| Malignancy | Defined from diagnoses of cancer (non-benign) | ICD-10: C00-C97 |
| Clinical outcomes |  |  |
| Coronary heart disease | Defined from an event of acute myocardial infarction, coronary revascularization, or death of which the cause was recorded as a coronary artery disease or myocardial infarction | Acute myocardial infarction: admission diagnosis (ICD-10: I21, I22) concurrently with coronary angiography (HA670, HA680, HA681)  Coronary revascularization: percutaneous coronary intervention (M6551, M6552, M6561, M6563, M6562, M6564, M6571, M6572), thrombolytic treatment (M6634), or coronary artery bypass graft (O1641, OA641, O1642, OA642, O1647, OA647)  Coronary artery disease or myocardial infarction:  ICD-10: I20, I21, I22, I23, I25 |
| Ischemic stroke^1,3^ | Defined from any discharge diagnoses with concomitant imaging studies | ICD-10: I63, I64 |
| Systemic embolism | Defined from admission diagnosis or related death | ICD-10: I74, N280 (including renal infarction) |

Abbreviations: eGFR, estimated glomerular filtration rate; ICD-10, International Classification of Diseases-10th Revision. *To ensure accuracy, comorbidities were established based on one inpatient or two outpatient records of ICD-10 codes in the database.

Supplementary Table 2. Risk of all-cause mortality, cardiovascular mortality, and non-cardiovascular disease related mortality according to physical activity among older adults with heart failure.

|  | Patients (n) | Events (n) | Events, /100 PYR | Unadjusted HR  (95% CI) | *p* value | Adjusted HR  (95% CI) | *p* value |
| --- | --- | --- | --- | --- | --- | --- | --- |
| All-cause mortality | | | | | | | |
| Female | | | | | | | |
| Inactive | 3009 | 532 | 6.0 | Reference |  | Reference |  |
| Insufficiently active | 1577 | 172 | 3.5 | 0.58 (0.49–0.68) | <0.001 | 0.81 (0.67–0.97) | 0.023 |
| Active | 1192 | 103 | 2.7 | 0.45 (0.37–0.56) | <0.001 | 0.79 (0.64–0.99) | 0.039 |
| Highly active | 559 | 33 | 1.8 | 0.30 (0.21–0.43) | <0.001 | 0.58 (0.40–0.84) | 0.004 |
| Male | | | | | | | |
| Inactive | 977 | 244 | 9.3 | Reference |  | Reference |  |
| Insufficiently active | 577 | 111 | 6.5 | 0.70 (0.56–0.87) | 0.002 | 0.81 (0.64–1.03) | 0.083 |
| Active | 633 | 115 | 5.9 | 0.63 (0.51–0.78) | <0.001 | 0.85 (0.68–1.07) | 0.162 |
| Highly active | 508 | 49 | 3.0 | 0.32 (0.23–0.43) | <0.001 | 0.50 (0.36–0.68) | <0.001 |
| CVD mortality | | | | | | | |
| Female | | | | | | | |
| Inactive | 3009 | 189 | 6.4 | Reference |  | Reference |  |
| Insufficiently active | 1577 | 63 | 3.8 | 0.60 (0.45–0.79) | <0.001 | 0.91 (0.67–1.23) | 0.533 |
| Active | 1192 | 41 | 3.2 | 0.51 (0.36–0.71) | <0.001 | 0.94 (0.66–1.34) | 0.743 |
| Highly active | 559 | 15 | 2.5 | 0.39 (0.23–0.65) | <0.001 | 0.84 (0.48–1.46) | 0.541 |
| Male | | | | | | | |
| Inactive | 927 | 72 | 8.2 | Reference |  | Reference |  |
| Insufficiently active | 577 | 32 | 5.6 | 0.68 (0.45–1.03) | 0.069 | 0.89 (0.59–1.34) | 0.581 |
| Active | 633 | 36 | 5.5 | 0.67 (0.45–0.99) | 0.046 | 0.97 (0.64–1.48) | 0.895 |
| Highly active | 508 | 15 | 2.8 | 0.33 (0.19–0.58) | <0.001 | 0.57 (0.32–1.00) | 0.052 |
| Non-CVD-related mortality | | | | | | | |
| Female | | | | | | | |
| Inactive | 3009 | 343 | 3.9 | Reference |  | Reference |  |
| Insufficiently active | 1577 | 109 | 2.2 | 0.57 (0.46–0.70) | <0.001 | 0.76 (0.60–0.95) | 0.017 |
| Active | 1192 | 62 | 1.6 | 0.42 (0.32–0.55) | <0.001 | 0.72 (0.54–0.95) | 0.022 |
| Highly active | 559 | 18 | 1.0 | 0.25 (0.16–0.41) | <0.001 | 0.45 (0.28–0.74) | 0.002 |
| Male | | | | | | | |
| Inactive | 927 | 172 | 6.5 | Reference |  | Reference |  |
| Insufficiently active | 577 | 79 | 4.6 | 0.71 (0.54–0.92) | 0.010 | 0.78 (0.59–1.03) | 0.085 |
| Active | 633 | 79 | 4.0 | 0.61 (0.47–0.80) | <0.001 | 0.80 (0.61–1.05) | 0.112 |
| Highly active | 508 | 34 | 2.1 | 0.31 (0.22–0.45) | <0.001 | 0.47 (0.32–0.68) | <0.001 |

HR, **[hazard ratio](https://en.wikipedia.org/wiki/Hazard_ratio)**; CI, confidence interval; CVD, cardiovascular disease; PYR**,** person-years at risk. The model was adjusted for age, sex, body mass index, hypertension, diabetes, dyslipidemia, chronic kidney disease, chronic obstructive pulmonary disease, malignancy, previous myocardial infarction, peripheral artery disease, vascular disease, prior stroke or transient ischemic attack, osteoporosis, Hospital Frailty Risk Score, Hospital Frailty Risk Score category, Charlson Comorbidity Index, smoking, and alcohol drinking.

Supplementary Table 3. Risk of hospitalization due to all-cause and heart failure, cardiovascular disease and stroke according to physical activity among older adults with heart failure.

|  | Patients (n) | Events (n) | Events, /100 PYR | Unadjusted HR (95% CI) | *p* value | Adjusted HR (95% CI) | *p* value |
| --- | --- | --- | --- | --- | --- | --- | --- |
| **Hospitalization due to all-cause** | | | | | | | |
| Female | | | | | | | |
| Inactive | 3009 | 1989 | 39.3 | Reference |  | Reference |  |
| Insufficiently active | 1577 | 1022 | 35.3 | 0.90 (0.84–0.98) | 0.010 | 0.97 (0.89–1.05) | 0.403 |
| Active | 1192 | 743 | 32.4 | 0.83 (0.77–0.91) | <0.001 | 0.93 (0.85–1.01) | 0.103 |
| Highly active | 559 | 332 | 29.1 | 0.75 (0.67–0.84) | <0.001 | 0.83 (0.74–0.93) | 0.002 |
| Male | | | | | | | |
| Inactive | 927 | 624 | 41.3 | Reference |  | Reference |  |
| Insufficiently active | 577 | 378 | 37.7 | 0.92 (0.80–1.04) | 0.177 | 0.98 (0.86–1.12) | 0.768 |
| Active | 633 | 413 | 34.4 | 0.85 (0.75–0.96) | 0.010 | 0.96 (0.85–1.09) | 0.545 |
| Highly active | 508 | 287 | 26.7 | 0.67 (0.59–0.78) | <0.001 | 0.80 (0.69–0.93) | 0.003 |
| **Hospitalization due to heart failure** | | | | | | | |
| Female | | | | | | | |
| Inactive | 3009 | 232 | 2.7 | Reference |  | Reference |  |
| Insufficiently active | 1577 | 103 | 2.1 | 0.80 (0.63–1.00) | 0.055 | 0.94 (0.75–1.20) | 0.640 |
| Active | 1192 | 64 | 1.7 | 0.65 (0.49–0.85) | 0.002 | 0.84 (0.63–1.12) | 0.231 |
| Highly active | 559 | 26 | 1.5 | 0.54 (0.42–0.76) | 0.003 | 0.71 (0.46–1.07) | 0.104 |
| Male | | | | | | | |
| Inactive | 927 | 55 | 2.1 | Reference |  | Reference |  |
| Insufficiently active | 577 | 43 | 2.6 | 1.22 (0.82–1.82) | 0.320 | 1.38 (0.92–2.07) | 0.119 |
| Active | 633 | 43 | 2.3 | 1.06 (0.71–1.58) | 0.780 | 1.31 (0.87–1.98) | 0.202 |
| Highly active | 508 | 23 | 1.4 | 0.67 (0.41–1.09) | 0.108 | 0.90 (0.54–1.48) | 0.667 |
| **Cardiovascular disease** | | | | | | | |
| Female | | | | | | | |
| Inactive | 3009 | 100 | 1.1 | Reference |  | Reference |  |
| Insufficiently active | 1577 | 52 | 1.1 | 0.93 (0.67–1.30) | 0.681 | 1.07 (0.76–1.51) | 0.690 |
| Active | 1192 | 39 | 1.0 | 0.92 (0.63–1.33) | 0.640 | 1.14 (0.77–1.68) | 0.515 |
| Highly active | 559 | 16 | 0.9 | 0.78 (0.46–1.32) | 0.352 | 1.03 (0.60–1.77) | 0.912 |
| Male | | | | | | | |
| Inactive | 927 | 59 | 2.3 | Reference |  | Reference |  |
| Insufficiently active | 577 | 27 | 1.6 | 0.71 (0.45–1.11) | 0.134 | 0.71 (0.45–1.13) | 0.152 |
| Active | 633 | 39 | 2.0 | 0.90 (0.60–1.34) | 0.591 | 0.97 (0.64–1.48) | 0.899 |
| Highly active | 508 | 35 | 2.2 | 0.97 (0.64–1.48) | 0.893 | 1.07 (0.70–1.64) | 0.744 |
| **Stroke** | | | | | | | |
| Female | | | | | | | |
| Inactive | 3009 | 170 | 2.0 | Reference |  | Reference |  |
| Insufficiently active | 1577 | 87 | 1.8 | 0.92 (0.71–1.19) | 0.534 | 1.04 (0.80–1.35) | 0.794 |
| Active | 1192 | 63 | 1.7 | 0.87 (0.65–1.15) | 0.324 | 1.02 (0.76–1.38) | 0.885 |
| Highly active | 559 | 20 | 1.1 | 0.57 (0.36–0.90) | 0.016 | 0.72 (0.45–1.15) | 0.169 |
| Male | | | | | | | |
| Inactive | 927 | 76 | 3.0 | Reference |  | Reference |  |
| Insufficiently active | 577 | 48 | 2.9 | 0.97 (0.68–1.40) | 0.882 | 1.00 (0.69–1.45) | 0.991 |
| Active | 633 | 45 | 2.4 | 0.78 (0.54–1.13) | 0.187 | 0.80 (0.55–1.17) | 0.247 |
| Highly active | 508 | 30 | 1.9 | 0.62 (0.41–0.94) | 0.026 | 0.67 (0.43–1.03) | 0.069 |

HR, **[hazard ratio](https://en.wikipedia.org/wiki/Hazard_ratio);** CI, confidence interval; PYR, person-years at risk.

The model was adjusted for age, sex, body mass index, hypertension, diabetes, dyslipidemia, chronic kidney disease, chronic obstructive pulmonary disease, malignancy, previous myocardial infarction, peripheral artery disease, vascular disease, prior stroke or transient ischemic attack, osteoporosis, Hospital Frailty Risk Score, Hospital Frailty Risk Score category, Charlson Comorbidity Index, smoking, and alcohol drinking.

Supplementary Table 4. Risk of all-cause mortality, cardiovascular mortality, and non-cardiovascular disease related mortality according to physical activity among older adults with heart failure.

|  | Patients (n) | Events (n) | Events, /100 PYR | Unadjusted HR (95% CI) | *p* value | Adjusted HR (95% CI) | *p* value |
| --- | --- | --- | --- | --- | --- | --- | --- |
| All-cause mortality | | | | | | | |
| HF medication group I - none or ACE inhibitor/ARB | | | | | | | |
| Inactive | 152 | 32 | 7.2 | Reference |  | Reference |  |
| Insufficiently active | 82 | 11 | 4.6 | 0.65 (0.33–1.28) | 0.211 | 1.26 (0.53–3.00) | 0.600 |
| Active | 73 | 1 | 4.2 | 0.06 (0.01–0.44) | 0.006 | 0.15 (0.02–1.19) | 0.070 |
| Highly active | 43 | 2 | 1.4 | 0.20 (0.05–0.81) | 0.025 | 0.29 (0.06–1.37) | 0.120 |
| HF medication group II - beta blocker or diuretics | | | | | | | |
| Inactive | 1880 | 309 | 5.6 | Reference |  | Reference |  |
| Insufficiently active | 1104 | 106 | 3.1 | 0.54 (0.44–0.68) | <0.001 | 0.72 (0.57–0.91) | 0.010 |
| Active | 998 | 83 | 2.6 | 0.46 (0.36–0.59) | <0.001 | 0.68 (0.53–0.87) | <0.001 |
| Highly active | 617 | 35 | 1.7 | 0.31 (0.22–0.44) | <0.001 | 0.45 (0.31–0.65) | <0.001 |
| HF medication group III - spironolactone | | | | | | | |
| Inactive | 1904 | 435 | 7.8 | Reference |  | Reference |  |
| Insufficiently active | 968 | 166 | 5.6 | 0.71 (0.60–0.85) | <0.001 | 0.87 (0.72–1.05] | 0.140 |
| Active | 754 | 134 | 5.8 | 0.74 (0.61–0.90) | 0.002 | 0.97 (0.8–1.19] | 0.790 |
| Highly active | 407 | 45 | 3.4 | 0.43 (0.32–0.59) | <0.001 | 0.62 (0.45–0.86] | <0.001 |
| CVD mortality | | | | | | | |
| HF medication group I - none or ACE inhibitor/ARB | | | | | | | |
| Inactive | 152 | 6 | 40.4 | Reference |  | Reference |  |
| Insufficiently active | 82 | 3 | 37.7 | 0.96 (0.24–3.82) | 0.958 | 1.88(0.44–8.07) | 0.400 |
| Active | 73 | 0 | 0 | 0.00 (0.00–0.00) | <0.001 | 0(0–0) | <0.001 |
| Highly active | 43 | 1 | 20.8 | 0.54 (0.07–4.37) | 0.564 | 1.71(0.19–15.39) | 0.630 |
| HF medication group II - beta blocker or diuretics | | | | | | | |
| Inactive | 1880 | 102 | 55.5 | Reference |  | Reference |  |
| Insufficiently active | 1104 | 31 | 26.8 | 0.48 (0.32–0.72) | <0.001 | 0.73 (0.49–1.1) | 0.140 |
| Active | 998 | 24 | 22.5 | 0.40 (0.26–0.63) | <0.001 | 0.72 (0.44–1.16) | 0.180 |
| Highly active | 617 | 11 | 16.5 | 0.30 (0.16–0.55) | <0.001 | 0.54 (0.28–1.03) | 0.060 |
| HF medication group III - spironolactone | | | | | | | |
| Inactive | 1904 | 153 | 82.4 | Reference |  | Reference |  |
| Insufficiently active | 968 | 61 | 61.6 | 0.74 (0.55–1.00) | 0.050 | 0.98 (0.72–1.33) | 0.900 |
| Active | 754 | 53 | 69 | 0.83 (0.61–1.14) | 0.253 | 1.14 (0.82–1.58) | 0.450 |
| Highly active | 407 | 18 | 41.2 | 0.50 (0.30–0.81) | 0.005 | 0.79 (0.47–1.34) | 0.380 |
| Non-CVD-related mortality | | | | | | | |
| HF medication group I - none or ACE inhibitor/ARB | | | | | | | |
| Inactive | 152 | 26 | 5.8 | Reference |  | Reference |  |
| Insufficiently active | 82 | 8 | 3.4 | 0.58 (0.26–1.27) | 0.173 | 0.99 (0.34–2.85) | 0.980 |
| Active | 73 | 1 | 4.2 | 0.07 (0.01–0.55) | 0.011 | 0.16 (0.02–1.71) | 0.130 |
| Highly active | 43 | 1 | 6.9 | 0.12 (0.02–0.90) | 0.039 | 0.19 (0.02–1.42) | 0.110 |
| HF medication group II - beta blocker or diuretics | | | | | | | |
| Inactive | 1880 | 207 | 3.8 | Reference |  | Reference |  |
| Insufficiently active | 1104 | 75 | 2.2 | 0.57 (0.44–0.75) | <0.001 | 0.71 (0.54–0.94) | 0.020 |
| Active | 998 | 59 | 1.8 | 0.49 (0.37–0.65) | <0.001 | 0.66 (0.49–0.9) | 0.010 |
| Highly active | 617 | 24 | 1.2 | 0.32 (0.21–0.48) | <0.001 | 0.42 (0.27–0.65) | <0.001 |
| HF medication group III - spironolactone | | | | | | | |
| Inactive | 1904 | 282 | 5.6 | Reference |  | Reference |  |
| Insufficiently active | 968 | 105 | 3.5 | 0.70 (0.56–0.87) | 0.002 | 0.81 (0.64–1.02) | 0.080 |
| Active | 754 | 81 | 3.5 | 0.69 (0.54–0.88) | 0.003 | 0.89 (0.69–1.15) | 0.360 |
| Highly active | 407 | 27 | 2.1 | 0.40 (0.27–0.60) | <0.001 | 0.54 (0.36–0.82) | <0.001 |

HR, **[hazard ratio](https://en.wikipedia.org/wiki/Hazard_ratio)**; CI, confidence interval; CVD, cardiovascular disease; PYR, person-years at risk; HF, heart failure. The model was adjusted for age, sex, body mass index, hypertension, diabetes, dyslipidemia, chronic kidney disease, chronic obstructive pulmonary disease, malignancy, previous myocardial infarction, peripheral artery disease, vascular disease, prior stroke or transient ischemic attack, osteoporosis, Hospital Frailty Risk Score, Hospital Frailty Risk Score category, Charlson Comorbidity Index, smoking, and alcohol drinking.

Supplementary Table 5. Risk of hospitalization due to all-cause and heart failure, cardiovascular disease and stroke according to physical activity among older adults with heart failure.

|  | Patients (n) | Events (n) | Events, /100 PYR | Unadjusted HR (95% CI) | *p* value | Adjusted HR (95% CI) | *p* value |
| --- | --- | --- | --- | --- | --- | --- | --- |
| Hospitalization due to all-cause | | | | | | | |
| HF medication group I - none or ACE inhibitor/ARB | | | | | | | |
| Inactive | 152 | 85 | 29.3 | Reference |  | Reference |  |
| Insufficiently active | 82 | 43 | 27.3 | 0.90 (0.62–1.31) | 0.592 | 1.03 (0.68–1.56) | 0.890 |
| Active | 73 | 34 | 19.6 | 0.67 (0.46–0.97) | 0.032 | 0.78 (0.52–1.16) | 0.220 |
| Highly active | 43 | 15 | 13.8 | 0.50 (0.28–0.89) | 0.018 | 0.52 (0.28–0.94) | 0.030 |
| HF medication group II - beta blocker or diuretics | | | | | | | |
| Inactive | 1880 | 1174 | 35.3 | Reference |  | Reference |  |
| Insufficiently active | 1104 | 671 | 31.3 | 0.89 (0.81–0.98) | 0.022 | 0.95 (0.86–1.05) | 0.310 |
| Active | 998 | 586 | 28.8 | 0.83 (0.75–0.92) | <0.001 | 0.92 (0.83–1.02) | 0.100 |
| Highly active | 617 | 331 | 24.7 | 0.72 (0.63–0.81) | <0.001 | 0.79 (0.7–0.9) | <0.001 |
| HF medication group III - spironolactone | | | | | | | |
| Inactive | 1904 | 1354 | 45.9 | Reference |  | Reference |  |
| Insufficiently active | 968 | 686 | 43.0 | 0.94 (0.86–1.03) | 0.199 | 1 (0.91–1.1) | 0.980 |
| Active | 754 | 536 | 41.6 | 0.91 (0.83–1.01) | 0.071 | 0.99 (0.9–1.1) | 0.890 |
| Highly active | 407 | 273 | 35.5 | 0.80 (0.70–0.91) | 0.001 | 0.87 (0.76–1) | 0.050 |
| Hospitalization due to heart failure | | | | | | | |
| HF medication group I - none or ACE inhibitor/ARB | | | | | | | |
| Inactive | 152 | 1 | 2.3 | Reference |  | Reference |  |
| Insufficiently active | 82 | 1 | 4.2 | 1.79 (0.11–28.76) | 0.680 | 1.86 (0.17–20.1) | 0.610 |
| Active | 73 | 0 | 0.0 | 0.00 (0.00–0.00) | <0.001 | 0.00 (0.00–0.00) | <0.001 |
| Highly active | 43 | 0 | 0.0 | 0.00 (0.00–0.00) | <0.001 | 0.00 (0.00–0.00) | <0.001 |
| HF medication group II - beta blocker or diuretics | | | | | | | |
| Inactive | 1880 | 59 | 1.1 | Reference |  | Reference |  |
| Insufficiently active | 1104 | 27 | 0.8 | 0.73 (0.46–1.15) | 0.172 | 0.84 (0.53–1.33) | 0.460 |
| Active | 998 | 28 | 0.9 | 0.82 (0.52–1.29) | 0.394 | 1.01 (0.63–1.62) | 0.960 |
| Highly active | 617 | 12 | 0.6 | 0.56 (0.30–1.04) | 0.065 | 0.67 (0.35–1.26) | 0.210 |
| HF medication group III - spironolactone | | | | | | | |
| Inactive | 1904 | 227 | 4.3 | Reference |  | Reference |  |
| Insufficiently active | 968 | 118 | 4.2 | 0.98 (0.79–1.23) | 0.883 | 1.12 (0.89–1.41) | 0.320 |
| Active | 754 | 79 | 3.6 | 0.84 (0.65–1.08) | 0.167 | 0.99 (0.76–1.29) | 0.960 |
| Highly active | 407 | 37 | 2.9 | 0.68 (0.48–0.97) | 0.032 | 0.84 (0.58–1.22) | 0.360 |
| Cardiovascular disease | | | | | | | |
| HF medication group I - none or ACE inhibitor/ARB | | | | | | | |
| Inactive | 152 | 1 | 2.3 | Reference |  | Reference |  |
| Insufficiently active | 82 | 2 | 0.8 | 3.54 (0.33–38.27) | 0.297 | 0.00 (0.00–0.00) | <0.001 |
| Active | 73 | 1 | 0.4 | 1.85 (0.11–31.25) | 0.671 | 0.03 (0.01–0.12) | <0.001 |
| Highly active | 43 | 0 | 0.0 | 0.00 (0.00–0.00) | <0.001 | 0.00 (0.00–0.00) | <0.001 |
| HF medication group II - beta blocker or diuretics | | | | | | | |
| Inactive | 1880 | 64 | 1.2 | Reference |  | Reference |  |
| Insufficiently active | 1104 | 29 | 0.8 | 0.72 (0.47–1.12) | 0.146 | 0.75 (0.48–1.17) | 0.210 |
| Active | 998 | 31 | 1.0 | 0.84 (0.55–1.29) | 0.434 | 0.85 (0.54–1.32) | 0.460 |
| Highly active | 617 | 24 | 1.2 | 1.04 (0.65–1.66) | 0.862 | 0.98 (0.61–1.59) | 0.940 |
| HF medication group III - spironolactone | | | | | | | |
| Inactive | 1904 | 94 | 1.7 | Reference |  | Reference |  |
| Insufficiently active | 968 | 48 | 1.7 | 0.96 (0.68–1.36) | 0.821 | 1.01 (0.71–1.45) | 0.950 |
| Active | 754 | 46 | 2.0 | 1.19 (0.84–1.69) | 0.338 | 1.19 (0.82–1.74) | 0.350 |
| Highly active | 407 | 27 | 2.1 | 1.24 (0.81–1.91) | 0.325 | 1.29 (0.83–2.02) | 0.260 |
| Stroke | | | | | | | |
| HF medication group I - none or ACE inhibitor/ARB | | | | | | | |
| Inactive | 152 | 5 | 1.1 | Reference |  | Reference |  |
| Insufficiently active | 82 | 2 | 0.9 | 0.70 (0.14–3.55) | 0.667 | 0.39 (0.04–3.46) | 0.390 |
| Active | 73 | 1 | 0.4 | 0.37 (0.04–3.17) | 0.362 | 0.42 (0.05–3.79) | 0.440 |
| Highly active | 43 | 2 | 1.4 | 1.25 (0.23–6.67) | 0.794 | 1.32 (0.09–20.51) | 0.840 |
| HF medication group II - beta blocker or diuretics | | | | | | | |
| Inactive | 1880 | 84 | 1.6 | Reference |  | Reference |  |
| Insufficiently active | 1104 | 65 | 1.9 | 1.24 (0.90–1.72) | 0.191 | 1.40 (1.00–1.95) | 0.050 |
| Active | 998 | 48 | 1.5 | 0.98 (0.69–1.40) | 0.924 | 1.12 (0.77–1.62) | 0.560 |
| Highly active | 617 | 22 | 1.1 | 0.72 (0.45–1.15) | 0.166 | 0.79 (0.49–1.28) | 0.330 |
| HF medication group III - spironolactone | | | | | | | |
| Inactive | 1904 | 157 | 2.9 | Reference |  | Reference |  |
| Insufficiently active | 968 | 68 | 2.4 | 0.81 (0.61–1.08) | 0.152 | 0.8 (0.6–1.07) | 0.130 |
| Active | 754 | 59 | 2.6 | 0.90 (0.67–1.21) | 0.484 | 0.87 (0.64–1.18) | 0.370 |
| Highly active | 407 | 26 | 2.0 | 0.69 (0.46–1.04) | 0.077 | 0.66 (0.43–1.01) | 0.060 |

HR, **[hazard ratio](https://en.wikipedia.org/wiki/Hazard_ratio)**; CI, confidence interval; CVD, cardiovascular disease; PYR**,** person-years at risk; HF, heart failure. The model was adjusted for age, sex, body mass index, hypertension, diabetes, dyslipidemia, chronic kidney disease, chronic obstructive pulmonary disease, malignancy, previous myocardial infarction, peripheral artery disease, vascular disease, prior stroke or transient ischemic attack, osteoporosis, Hospital Frailty Risk Score, Hospital Frailty Risk Score category, Charlson Comorbidity Index, smoking, and alcohol drinking.
